# Retraction Note: Exploring the Burden of Cancer in Pakistan: an analysis of 2019 Data

**DOI:** 10.1007/s44197-025-00483-x

**Published:** 2025-11-03

**Authors:** Muhammad Tufail, Changxin Wu

**Affiliations:** Institute of Biomedical Sciences, Shanxi University, Taiyuan, 030006 China

**Retraction Note: Journal of Epidemiology and Global Health (2023) 13:333-343**

10.1007/s44197-023-00104-5

The Editor-in-Chief has retracted this article because of a substantial content, data and image overlap with a previously-published and uncited PAEC report by different authors [1]. The authors did not state explicitly whether they agree to this retraction.

## References

[1] Sohaib M, Shafiq A, PAEC Cancer Registry. Report 2018 and 2019. Islamabad, Pakistan: Nuclear Medicine & Oncology Division Pakistan Atomic Energy Commission; 2019. Available at [+ https://paec.gov.pk/Documents/Medical/PAECR_report_2018-19.pdf +](last accessed on 27 July 2025).

